# Applied Comparison of Meta-analysis Techniques

**DOI:** 10.36469/9848

**Published:** 2013-02-28

**Authors:** Li Wang, Colin Lewis-Beck, Elyse Fritschel, Erdem Baser, Onur Baser

**Affiliations:** 1 STATinMED Research, Dallas, TX, USA; 2 Freddie Mac, Washington, DC, USA; 3 STATinMED Research, Istanbul, Turkey; 4 The University of Michigan and STATinMED Research, Ann Arbor, MI, USA

**Keywords:** meta-regression, random-effects model, fixed-effects model, tuberculosis, meta-analysis

## Abstract

**Background:** Meta-analysis is an approach that combines findings from similar studies. The aggregation of study level data can provide precise estimates for outcomes of interest, allow for unique treatment comparisons, and explain the differences arising from conflicting study results. Proper meta-analysis includes five basic steps: identify relevant studies; extract summary data from each paper; compute study effect sizes, perform statistical analysis; and interpret and report the results.

**Objectives:** This study aims to review meta-analysis methods and their assumptions, apply various meta-techniques to empirical data, and compare the results from each method.

**Methods:** Three different meta-analysis techniques were applied to a dataset looking at the effects of the bacille Calmette-Guerin (BCG) vaccine on tuberculosis (TB). First, a fixed-effects model was applied; then a random-effects model; and third meta-regression with study-level covariates were added to the model. Overall and stratified results, by geographic latitude were reported.

**Results:** All three techniques showed a statistically significant effects from the vaccination. However, once covariates were added, efficacy diminished. Independent variables, such as the latitude of the location in which the study was performed, appeared to be partially driving the results.

**Conclusions:** Meta-analysis is useful for drawing general conclusions from a variety of studies. However, proper study and model selection are important to ensure the correct interpretation of results. Basic meta-analysis models are fixed-effects, random-effects, and meta-regression.

## INTRODUCTION

An approach to systematically integrating and interpreting multiple analyses to develop a single conclusion was given the title 'meta-analysis' by Glass in 1976, and has been gaining popularity in a variety of research settings ever since.[1] As the quantity of studies aggregated increases, so does the precision in parameter estimation. Meta-analysis can provide confirmatory data analysis, extrapolate to a larger target population, develop new inferences by modeling the treatment variation across studies, and when comparing results from multiple randomized control trials (RCT), meta-analysis allows for the preservation of RCT design benefits of randomization on the effect size (ES).

The benefits associated with meta-analysis techniques are dependent on their proper implementation. The incorporation of information from a body of research into a model framework may help discover treatment moderators unobservable at the individual study level or generate misleading, biased results. For example, the exclusion of relevant studies or inclusion of inadequate studies through flawed selection strategies can lead to spurious conclusions, but can also be avoided through the use of systematic eligibility criteria.[2] Selected population, protocol, dosage levels, outcomes, lengths of follow-up, and other variables significantly correlated with outcomes should be delineated *a priori*.

After proper study selection, the data should then be collected and combined. Fixed-effect and random-effect models calculate weighted averages of results. However, fixed-effect models assume that variability between studies is exclusively due to random variation.[3] Additional techniques for calculating overall effect include multiple treatment comparisons, meta-regression, and subgroup analysis, each with underlying assumptions. Testing for heterogeneity, outliers, and investigating covariates that differ across studies should be analyzed before and after the model-fitting stage. Reporting the most representative statistics and figures are key to ensuring that results are transparent and accessible.

This research focuses on a set of studies concerning the effectiveness of the bacille Calmette-Guerin (BCG) vaccine on contraction of tuberculosis (TB). The BCG vaccine has been in use since 1921 and there have been numerous experiments studying its effectiveness. The results from some studies show a detrimental effect from the vaccine, while others found the vaccine to have an 80% benefit in reducing TB.[4] With each study as an observation, meta-analysis can be used to test the hypothesis that the BCG vaccine is effective against TB. Meta-analysis of BCG efficacy was conducted by Colditz *et al.* in 1994, using a random-effects model, and in this study, the incorporation of different meta-analysis techniques will be explored to help resolve the lack of consistency across study results.[4]

## METHODS

### Study Design

A retrospective analysis was performed using data from 13 trials on the efficacy of the BCG vaccine against TB from the Corditz *et al.* study (Table 1). The final 13 studies were selected based on inclusion/exclusion criteria to make the trials as homogeneous as possible. For example, only trials with randomized treatment and control groups were selected. Other important similarities included selection of studies with comparable follow-up duration and outcome variables preventing TB or death, rather than simply a medical reaction.

**Table 1.** Data Source[4]

| Trial | Author | Year Published | Treatment + | Treatment - | Control + | Control - | Absolute Latitude | Treatment Allocation |
|---|---|---|---|---|---|---|---|---|
| 1 | Aronson | 1948 | 4 | 119 | 11 | 128 | 44 | random |
| 2 | Fergunson & Simes | 1949 | 6 | 300 | 29 | 274 | 55 | random |
| 3 | Rosenthal *et al.* | 1960 | 3 | 228 | 11 | 209 | 42 | random |
| 4 | Hart & Sutherland | 1977 | 62 | 13536 | 248 | 12619 | 52 | random |
| 5 | Frimodt-Moller *et al.* | 1973 | 33 | 5036 | 47 | 5761 | 13 | alternate |
| 6 | Stein & Aronson | 1953 | 180 | 1361 | 372 | 1079 | 44 | alternate |
| 7 | Vandiviere *et al.* | 1973 | 8 | 2537 | 10 | 619 | 19 | random |
| 8 | TPT Madras | 1980 | 505 | 87886 | 499 | 87892 | 13 | random |
| 9 | Coetzee & Berjak | 1968 | 29 | 7470 | 45 | 7232 | 27 | random |
| 10 | Rosenthal *et al.* | 1961 | 17 | 1699 | 65 | 1600 | 42 | systematic |
| 11 | Comstock *et al.* | 1974 | 186 | 50448 | 141 | 27197 | 18 | systematic |
| 12 | Comstock & Webster | 1969 | 5 | 2493 | 3 | 2338 | 33 | systematic |
| 13 | Comstock *et al.* | 1976 | 27 | 16886 | 29 | 17825 | 33 | systematic |

In each trial, a vaccinated group was compared to a non-vaccinated control group. Each study was treated as one observation. Summary statistics from each study, such as latitude of study, method of randomization, and year of publication were used as covariates. Statistical analysis was performed using RevMan software (version 5, Cochrane Collaboration) and Winbugs software in a Bayesian Markov chain Monte Carlo (MCMC) framework.[5]

### Outcome Variables

For each trial, the odds ratio (OR) was calculated based on the number of subjects contracting TB in the treated and control populations. When disease incidence is rare (<10%), OR can approximate the relative risk (RR) in studies such as this.[6]

### Covariates

The following independent variable was tested:

- Absolute Latitude: The distance of each study from the equator in absolute value.[7]

## STATISTICAL ANALYSIS

Three different meta-analysis techniques were fitted to the BCG dataset. The most basic meta-analysis model (fixed-effects) was first used, followed by a random-effects model, and lastly meta-regression with study level covariates. Each of the three models are explained in more detail below.

### Fixed-effects Model

The population treatment effect, $\theta$, is assumed to be constant across studies. The only difference is due to sampling error. The model is:

$$y_i = \theta + \varepsilon_i \text{ where } \varepsilon_i \sim N(0, \sigma_i^2)$$

When calculating $\theta$, each trial is weighted by the inverse of its variance, which gives more weight to studies with higher precision, i.e. less variation:

$$w_i = 1/se(\theta_i)^2$$

Parameters are estimated using the Mantel-Haenszel method.[8]

### Random-effects Model

A random-effects model allows the true treatment effect to vary across studies. The observed ES is a combination of study-specific effects and the sampling error. Typically, the random distribution of treatment effects is assumed to follow a normal distribution.

$$y_i \mid \theta_i \sim N(\theta_i, \sigma_i^2) \text{ where } \theta_i \sim N(\theta, \tau^2)$$

Here, $\tau^2$ is the between-study variance. It is estimated from the data using the DerSimonian and Laird estimator.[9]

### Mixed-effects Model

Also known as meta-regression, mixed-effects models try to explain heterogeneities in the treatment effect by incorporating covariates into a linear regression model.

$$y_i \sim N(\theta_i, \sigma_i^2) \text{ where } \theta_i = x_i \beta$$

Here, $\beta$ is a vector of coefficients, and $x_i$ is a vector of covariate values for each study. Parameters are estimated using a restricted maximum-likelihood estimator. Due to the similarity between mean and median estimates, only means were provided in tabular form.

## RESULTS

### Fixed-effects Model (Table 2)

The OR generated from the fixed-effects model of all 13 selected trial studies indicated that the risk of contracting TB was reduced by 38% for the vaccinated group compared to the non-vaccinated group (OR= 0.62, 95% confidence interval [CI]: 0.57-0.68). Nearly all of the total variability in these studies was due to true heterogeneity (93%).

**Table 2.** Fixed-effects Model

| | Fixed-Effects Model | | | |
|---|---|---|---|---|
| | **Latitude ≥40** | **Latitude <40** | **Overall** | **Subgroup Differences** |
| **Chi²** | 10.78 | 21.43 | 163.94 | 138.95 |
| **I²** | 54% | 72% | 93% | 99.3% |
| **Overall effect: Z** | 15.66 | 2.14 | 11.54 | -- |
| **Overall effect: P** | <0.0001 | 0.03 | <0.0001 | <0.0001 |

Colditz *et al.* suspected that the influence of latitude on TB infection was the result of regional mycobacterial infections associated with geographic latitude. Additionally, since heat causes the drug to lose potency and direct exposure to sunlight could kill some of the bacteria required for the vaccine to work properly, it was expected that the drug would be more potent in colder climates. Hence, heterogeneity was tested using a binary measure of latitude (either above or below 40 degrees). The results are illustrated in the forest and funnel plots below (Figures 1-2).

**Figure 1.** Forest Plot - Fixed-effects Model

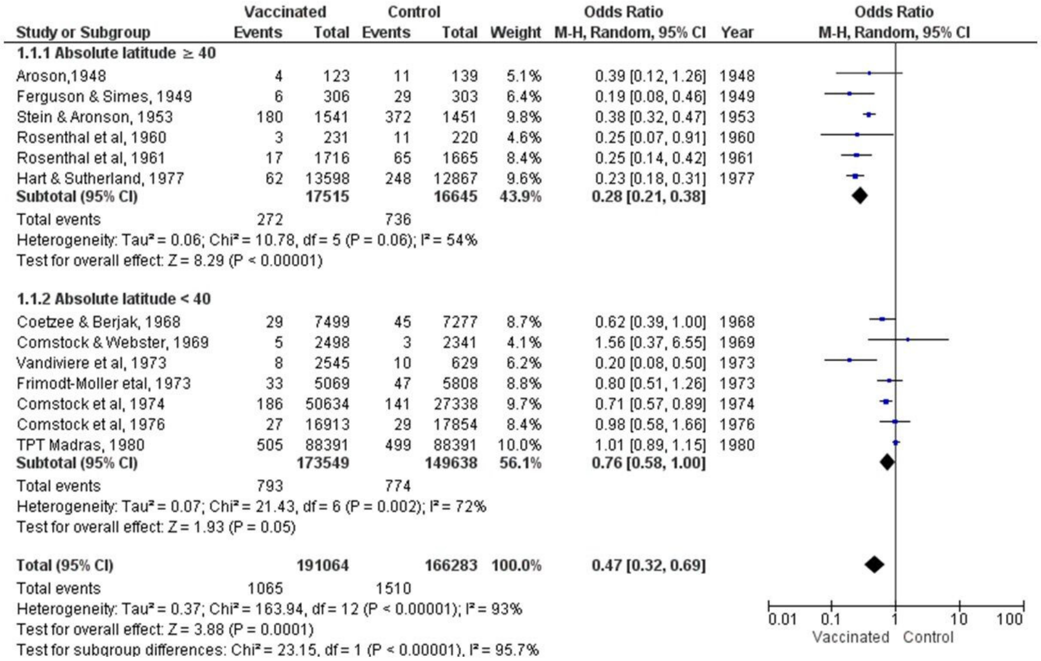

**Figure 2.** Fixed-effects Funnel Plot of Trial Studies by Latitude

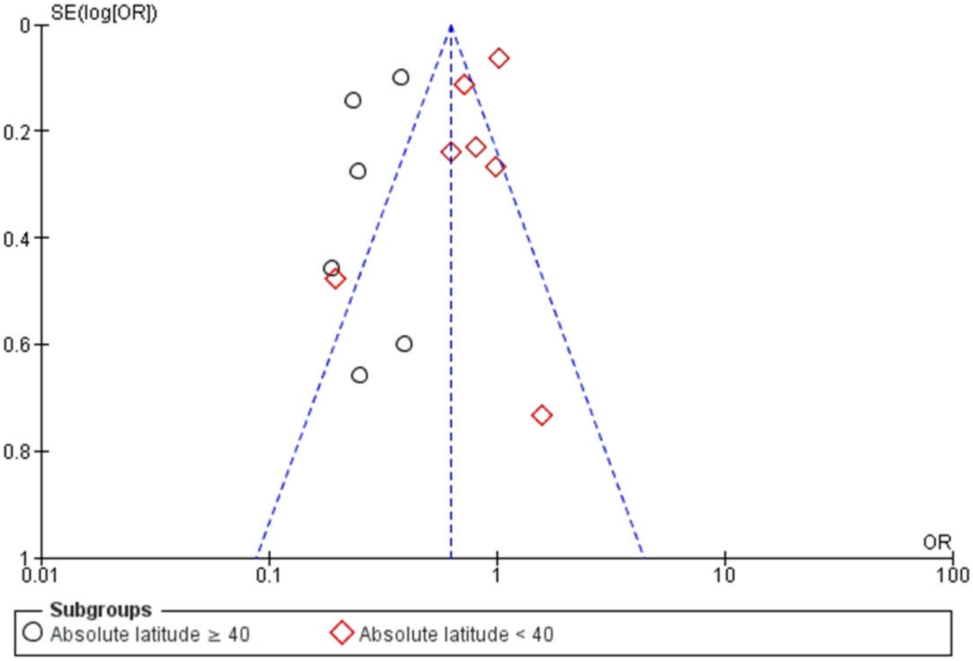

In studies located at or above the 40 degree latitude mark (N=6), the risk of contracting TB in the BCG-vaccinated group was 69% lower than in the non-vaccinated group (OR=0.31, 95% CI: 0.26-0.36). As suggested previously, these results confirm the stronger protective effect in higher latitudes with cooler climates.

Variability between studies (or true heterogeneity) reached 54%, which was a reduction from the heterogeneity found in all studies (93%). In studies located below the 40 degree latitude mark (N=7), the risk of contracting TB in the BCG-vaccinated group was 10% lower than in the non-vaccinated group (OR=0.90, 95% CI: 0.57-0.68) with 72% true heterogeneity.

**Random-effect Model (Table 3)**

A random-effects model was applied to account for treatment effect heterogeneity. After modeling the variation across studies, the overall treatment resulted in a stronger protective effect of 53% against TB in the vaccinated group (OR=0.47, 95% CI: 0.32-0.69) and between-study variance of 0.37.

As seen in the corresponding forest plot (Figure 3), studies located at a latitude of 40 degrees or above resulted in a strong protective effect (OR=0.28, 95% CI: 0.21-0.38) and between-study variance of 0.06. In studies located below 40 degrees, BCG vaccination still resulted in a protective effect against contracting TB, but the strength was diminished (OR=0.76, 95% CI: 0.58-1.00). Between-study variance reached 0.07. In the test for subgroup differences, true heterogeneity reached 95.7%, which reinforces the notion that these studies should be analyzed with stratification by geographic latitude.

**Table 3.** Random-effects Model

| | Random-Effects Model | | | |
|---|---|---|---|---|
| | **Latitude ≥40** | **Latitude <40** | **Overall** | **Subgroup Differences** |
| **$\tau^2$** | 0.06 | 0.07 | 0.37 | -- |
| **$Chi^2$** | -- | -- | -- | 23.15 |
| **$I^2$** | 54% | 72% | 93% | 95.7% |
| **Overall effect: Z** | 8.29 | 1.93 | 3.88 | -- |
| **Overall effect: P** | <0.0001 | 0.05 | <0.0001 | <0.0001 |

**Figure 3. Random-effects Forest Plot**

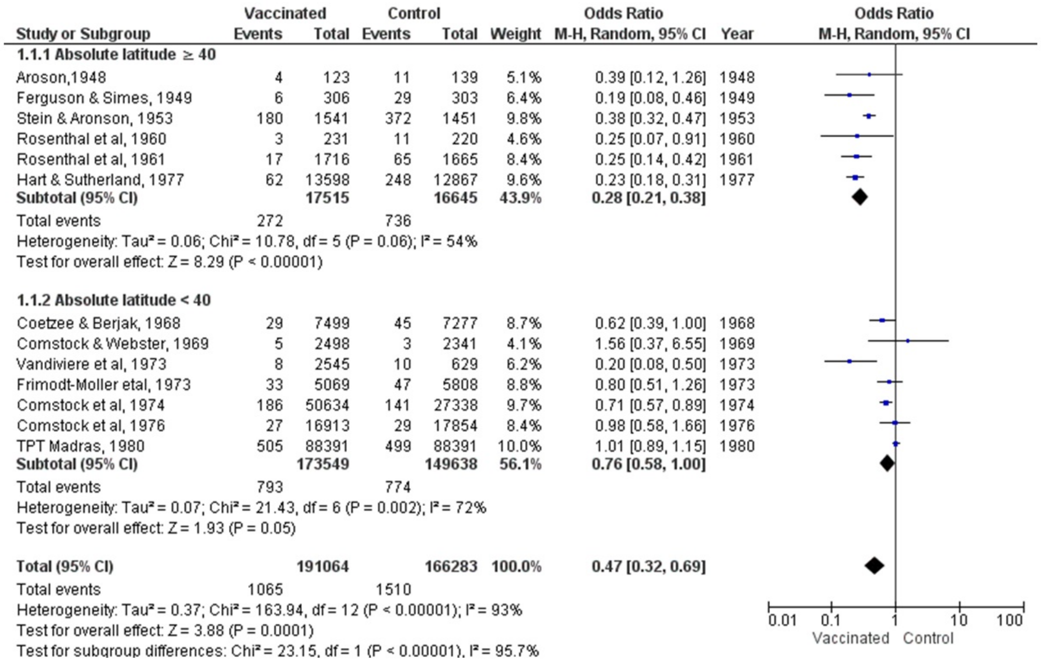

### Meta-regression (Table 4)

Lastly, absolute geographic latitude was added to the model as a covariate to explain differences in treatment effect across trials. Table 4 compares the results of meta-regression with and without the added latitude covariate covariate in a Bayesian MCMC framework.

**Table 4.** Meta-regression Results

| | No Covariates | | | | Added Latitude Covariate | | | |
|---|---|---|---|---|---|---|---|---|
| | Mean/Median | SD | 95% Credible Interval | | Mean/Median | SD | 95% Credible Interval | |
| OR | 0.477 | 0.105 | 0.299 | 0.712 | 0.788 | 0.128 | 0.537 | 1.049 |
| σ | 0.681 | 0.200 | 0.367 | 1.162 | 0.320 | 0.146 | 0.292 | 0.686 |
| Total Residual Deriviation | 26.04 | 7.196 | 13.62 | 41.88 | 27.2 | 6.926 | 15.24 | 42.06 |
| β | | | | | -1.024 | 0.255 | -1.533 | -0.517 |

Before adding latitude as a binary covariate, the OR generated from the meta-regression model indicated a significant protective effect of BCG vaccination on TB outcomes (OR=0.48, 95% credible interval: 0.47-0.71). The corresponding heterogeneity value was 0.68 with a posterior mean of total residual deviance value of 26.04.

When the latitude covariate was added, the OR of contracting TB indicated a protective effect. However, the credible interval indicated that the measure was not significant (OR=0.78, 95% credible interval: 0.54-1.05). In contrast, the coefficient of the latitude variable was statistically significant (β= -1.02, 95% credible interval: -1.53 to -0.52). As absolute latitude increased, or in studies further away from the equator, the risk of contracting TB decreased. The heterogeneity value decreased from 0.68 (without the added covariate) to 0.32, and the average total residual deviance reached an average/median of 27.2. Although the power of this study is limited by the quantity of included trials, a negative linear trend was observed: regardless of vaccination status, individuals further away from the equator were less likely to be diagnosed with TB.

## DISCUSSION

This review applied three different meta-analysis techniques to the BCG vaccine dataset. Each of the three methods produced slightly different parameter estimates of the overall vaccination effect. The fixed-effects and random-effects models generated protective effect measures of different strengths (OR: 0.62 [95% CI: 0.57-0.68] and 0.47 [95% CI: 0.32-0.69], respectively). When studies in fixed-effects and random-effects models were stratified by geographic latitude, the heterogeneity values were reduced, and as hypothesized previously, studies located at higher latitudes experienced a stronger protective vaccination effect against TB outcomes.

It is important to keep in mind that the goal of fixed effect analysis is to make a conditional inference pertaining only to the studies included, while the random-effects model allows for the unconditional inference of a larger hypothetical population. Although the random-effects model allows studies to vary in a normal distribution, it is not always the most appropriate tool when generalizing the results of a meta-analysis to real-world situations.[10]

The meta-regression model, which incorporated geographic latitude as a moderator of BCG vaccination, generated an effect measure denoting ambiguity in vaccination protection from TB outcomes (OR=0.78, [0.54-1.05]). The variation in protective effect measures seen in fixed-effects, random-effects, and meta-regression models highlights the influence of study design and adjustment on the interpretation of meta-analysis results. As one of the most widely used vaccines fighting the leading cause of human morbidity and mortality in the world, BCG implications are still medically relevant and well worth investigation.[11]

Meta-analysis is a relatively new field with the potential to observationally study a variety of evidence in a robust manner. Through the use of meta-analysis, hypotheses could be generated concerning the wide variation in treatment effectiveness in studies across the globe. However, meta-analysis has its own set of limitations that researchers should consider. For example, studies should be 'similar enough' to be aggregated together into a meta-analysis. While each study will not be methodologically or demographically identical, comparing studies that are too disparate will produce spurious results. There are no hard rules on setting inclusion/exclusion criteria, but the researcher should be able to justify the selected studies based on the question of interest. As with all statistical modelling, no statistical technique can correct for bad data. A retrospective analysis is limited to the data provided in the selected source papers. Information may be lacking concerning the original population of subjects and/or the study protocol. If the unobserved data is related to the contraction of TB, it would limit the generalizability of study results.

The process of fitting a regression model comes with additional limitations. As in standard regression analysis, studies may differ in unobserved ways not controlled for in the model. This is especially problematic in meta-analysis, where some studies may not have the same covariate information as the rest.[12] Furthermore, even if comparable covariates were available, the quantity that can be incorporated into a model is typically limited by small study sizes. Estimating robust parameter estimates can be difficult with such a limited number of degrees of freedom.

The final major limitation of meta-analysis, particularly meta-regression, is aggregation bias or the ecological fallacy.[13] Ecological fallacy refers to the false assumption that a statistical association observed between aggregate level variables resembles the association between the same variables at the individual level. In the case of the BCG trials, only aggregated study level data was available. However, a more robust study would incorporate individual data to look and the relationship between vaccination treatment modifiers and the incidence of TB. Unfortunately, individual data is not always available, and thus researchers are sometimes forced to rely on using aggregate study data to make inferences about the effects on individuals.

## CONCLUSION

Meta-analysis is useful for drawing general conclusions from a variety of studies. However, proper study and model selection are important to ensure the correct interpretation of results. The most basic meta-analysis models are fixed-effects, random-effects, and meta-regression which can generate a wide range of effect measures. Regardless of the specific model technique used, however, it is important to consider the overall meta-analysis process: properly selecting the studies, specifying the correct model, and translating the results from aggregate level data down to individual patients. Meta-analysis is a useful tool as it leverages previously collected data to make new causal assumptions between treatments and outcomes. However, care must be taken to make valid conclusions from the selected studies.

## CONFLICT OF INTEREST DECLARATION

The authors declare that they have no competing interests.
